# Tumor-associated mesenchymal stem-like cells provide extracellular signaling cue for invasiveness of glioblastoma cells

**DOI:** 10.18632/oncotarget.13638

**Published:** 2016-11-26

**Authors:** Eun-Jung Lim, Yongjoon Suh, Ki-Chun Yoo, Ji-Hyun Lee, In-Gyu Kim, Min-Jung Kim, Jong Hee Chang, Seok-Gu Kang, Su-Jae Lee

**Affiliations:** ^1^ Department of Life Science, Research Institute for Natural Sciences, Hanyang University, Seoul 04763, Republic of Korea; ^2^ Department of Neurosurgery, Brain Tumor Center, Severance Hospital, Yonsei University College of Medicine, Seoul 03722, Republic of Korea; ^3^ Department of Radiation Biology, Environmental Radiation Research Group, Korea Atomic Energy Research Institute, Daejeon 34057, Republic of Korea; ^4^ Laboratory of Radiation Exposure & Therapeutics, National Radiation Emergency Medical Center, Korea Institute of Radiological & Medical Sciences, Seoul 01812, Republic of Korea

**Keywords:** extracellular matrix remodeling, mesenchymal stem-like cells, hyaluronic acid, hyaluronic acid synthase-2, C5a

## Abstract

Hyaluronic acid (HA) is abundant in tumor microenvironment and closely associated with invasiveness of glioblastoma (GBM) cells. However, the cellular mechanism underlying HA-rich microenvironment in GBM remains unexplored. Here, we show that tumor-associated mesenchymal stem-like cells (tMSLCs) contribute to abundance of hyaluronic acid (HA) in tumor microenvironment through HA synthase-2 (HAS2) induction, and thereby enhances invasiveness of GBM cells. In an autocrine manner, C5a secreted by tMSLCs activated ERK MAPK for HAS2 induction in tMSLCs. Importantly, HA acted as a signaling ligand of its cognate receptor RHAMM for intracellular signaling activation underlying invasiveness of GBM cells. Taken together, our study suggests that tMSLCs contribute to HA-rich proinvasive ECM microenvironment in GBM.

## INTRODUCTION

Glioblastoma (GBM) is the most common and lethal primary brain tumor in adults [1]. Despite modern surgical and medical treatments, GBM still remains an incurable brain disease. A major barrier to the effective treatment of GBM is the invasiveness of these cells into brain parenchyma. In a process of invasion, GBM cells interact with a variety of extracellular matrix (ECM) molecules. In recent years, much attention has been devoted to ECM that are associated with cancer progression [2–4]. Among ECM components, hyaluronic acid (HA) is abundant in brain than other tissues, and has shown to provide microenvironmental cues for infiltration of GBM cells [5]. In addition, HA is more rich in brain tumors than surrounding normal brain parenchyma and its abundance is strongly correlated with poor prognosis of GBM patients [6]. HA is a large, negatively charged, unbranched polymer composed of repeating disaccharides of glucuronic acid and N-acetylglucosamine. In normal brain, HA is the primary ECM components and is known to exert beneficial effects on tissue homeostasis, the biomechanical integrity and structure, due to its viscosity and ability to retain water [5]. In pathological conditions including tumors, HA was found to be more enriched in the injury sites than normal counterpart tissues [5]. HA abundance in brain tumors has been known to promote motility of GBM cells by providing mechanical stiffening and acting as a ligand for intracellular signal transduction through its cognate receptors such as such as cluster determinant 44 (CD44), receptor for hyalunonate-mediated motility (RHAMM), and intercellular adhesion molecule-1 (ICAM-1) [5, 7, 8]. In accordance, the abundance of HA been proportional to invasiveness of tumor cells [9]. Moreover, high levels of HA in stroma are associated with poor prognosis of the patients [10]. However, although its mechanistic and cellular signaling functions in linkage with invasiveness of glioma cells are relatively well studied, the cellular mechanisms underlying the abundance of HA in brain tumors remain largely unexplored.

Recently, tumor-associated mesenchymal stem-like cells (tMSLCs) are reported as stromal cells interacting with GBM cells and their potential role in tumor progression has received intensive attention [11, 12]. However, their role in brain tumor progression remains largely unknown. Moreover, their ultimate contribution to tumor progression still remains controversial whether tMSLCs play a role in cancer promotion or suppression [13–16]. Since those contrasting results could be caused by differences in their origin and/or type of tumor studied, we previously isolated tMSLCs from human GBM surgical specimens and found that their presence is closely correlated with the prognosis of patients [12]. Given that endogenous mesenchymal stem cells (MSCs) have a wound tropism to injury sites under pathological conditions such as inflammation and alters microenvironmental cues including ECM to repair damaged tissues [17], we speculated that the abundance of HA in GBM stroma is attributable to the ECM remodeling ability of tMSLCs.

In this study, we demonstrate that HA is highly produced in tMSLCs by autocrine loop, in which C5a stimulated extracellular ERK MAPK through C5a receptor-1 (C5aR1) for HA synthase (HAS)-2 induction. C5a is an N-terminal 74 amino acid fragment of the a-chain of the complement fifth component (C5), and is well known as a complement component that triggers degranulation of mast cells or neutrophils, enhancing their phagocytosis of pathogens [18]. In this study, our findings suggest that C5a acts as a microenvironmental cue activating ERK MAPK that promotes HA production in tMSLCs. Collectively, we demonstrate that tMSLCs interacting with tumor cells provide HA-rich proinvasive microenvironment in brain tumor.

## RESULTS

### tMSLCs enhances invasiveness of GBM cells by remodeling ECM microenvironment

**Figure 1:**
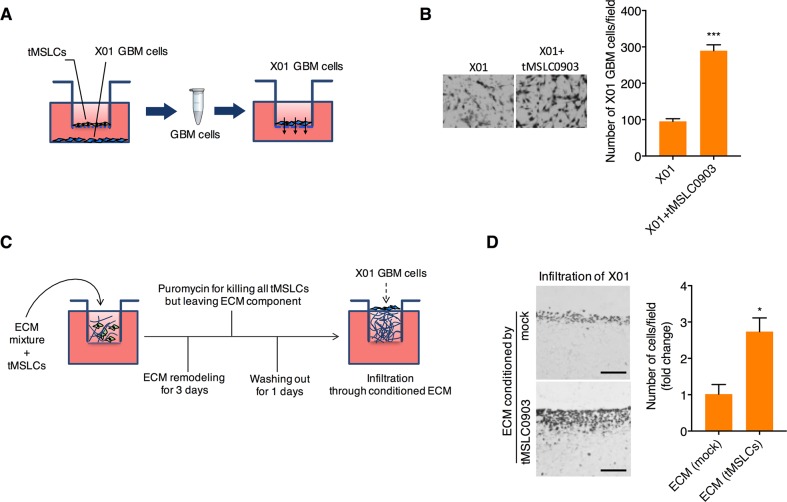
tMSLCs enhances invasiveness of GBM cells by remodeling ECM **A, B.** Schematic illustration (A) and quantification (B) of GBM cell migration after coculture with tMSLCs in transwells. **C.** Schematic illustrating the experimental scheme for analysis of GBM cell invasiveness in collagen-based ECM preconditioned by tMSLCs. **D.** H&E staining and quantification of X01 GBM cells infiltrated into the collagen-based matrix preconditioned by tMSLCs. Scale bar, 200 μm. Data are presented as mean ± SD from one of three independent experiments performed. *, p < 0.05 vs. control; ***, p < 0.001 vs. control.

To examine the effect of tMSLCs on invasiveness of GBM cells, we cocultured patient-derived X01 GBM cells with tMSLC0903 in Transwells. In this coculture, GBM cells were spaced from tMSLCs but crosstalk with each other through a porous membrane (Figure 1A). After coculture, invasiveness of GBM cells were analyzed in Transwells pre-coated with matrigel. By coculture with tMSLCs, X01 GBM cells adopted more invasiveness than non-cocultured cells (Figure 1B). In parallel, we also analyzed invasiveness of GBM cells in ECM conditioned by tMSLCs. To this end, we plated tMSLCs onto Millicell inserts containing a mixture of collagen type I and matrigel in growth medium, and allowed cells to remodel ECM for 3 d. All tMSLCs were then killed by treatment with puromycin, leaving ECM conditioned by tMSLCs, as depicted in Figure 1C. X01 GBM cells were then plated in this GBM cell-generated ECM, and their invasiveness was visualized by hematoxylin and eosin (H&E) staining after perpendicular sectioning of the gel matrix. X01 GBM cells in ECM conditioned by tMSLCs displayed greater infiltration than GBM cells plated on control ECM (Figure 1D). Taken together, these results suggest that tMSLCs promote invasiveness of GBM cells by providing extracellular components in tumor microenvironment.

### tMSLCs increases HA through HAS2 induction in GBM microenvironment

**Figure 2:**
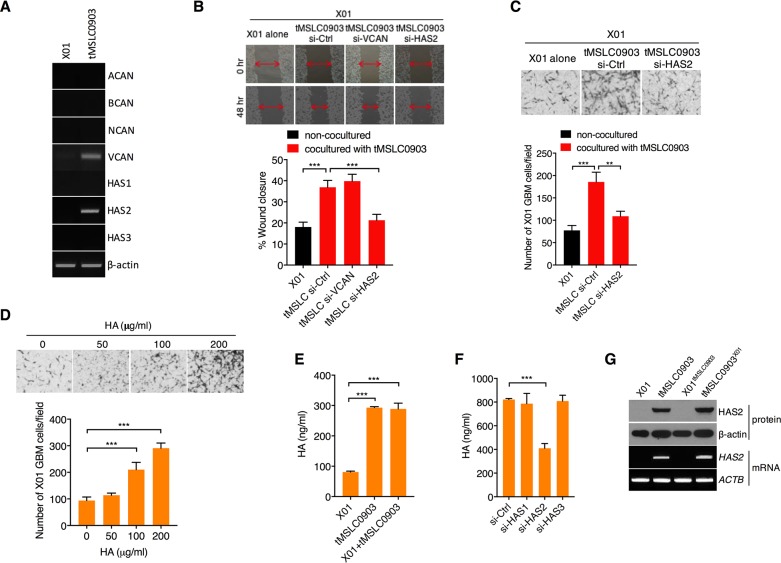
tMSLCs highly produce HA through HAS2 **A.** Semi-quantitative RT-PCR for ECM components and remodeling enzymes in X01 GBM cells and tMSLCs. **B.** Wound healing assay of X01 GBM cells alone or after coculture with tMSLCs transfected with siRNA against VCAN or HAS2. **C.** Migration of X01 GBM cells alone or after coculture with tMSLCs transfected with siRNA as indicated. **D.** Effect of HA on migration of X01 GBM cells in transwells. **E.** ELISA for HA levels in X01 GBM cells, tMSLCs or after coculture with tMSLC0903. **F.** ELISA for HA levels in tMSLC0903 after treatment with siRNA as indicated. **G.** Western blot and semi-quantitative RT-PCR for HAS2 levels in X01 GBM cells, tMSLCs or in coculture conditions. X01^tMSLC0903^ indicates X01 GBM cells cocultured with tMSLC0903, and tMSLC0903^X01^ indicates tMSLC0903 cocultured with X01 GBM cells. Data are presented as mean ± SD from one of three independent experiments performed. ***, p < 0.001 vs. control.

Given that tMSLCs change ECM composition for invasiveness of GBM cells, we next attempted to define the ECM components that are increased by tMSLCs and are responsible for invasiveness of GBM cells. By semi-quantitative RT-PCR, we analyzed the levels of ECM components that are mostly involved in GBM progression [19]. When those ECM components were compared between X01 GBM cells and tMSLCs, we found that HAS2 and Versican (VCAN) levels are particularly higher in tMSLCs than in GBM cells (Figure 2A). Accordingly, we depleted VCAN or HAS2 in tMSLCs and examined their coculture effect on migration of GBM cells. In wound healing assay, X01 GBM cells cocultured with tMSLCs more rapidly moved to fill the wound than non-cocultured cells; however, depletion of HAS2 abolished the effect of tMSLCs on migration of GBM cells (Figure 2B). Likewise, GBM cells were also more migratory in transwells when cocultured with tMSLCs; however, the effect of tMLSCs was diminished by transfection with small interfering RNA (siRNA) against HAS2, indicating that HAS2 is responsible for the effect of tMSLCs on migration of GBM cells (Figure 2C).

Since HAS2 is a rate limiting enzyme for HA synthesis, we next validated the effect of HA on migration of GBM cells. As expected, exogenous addition of HA enhanced migration of GBM cells in a dose dependent manner (Figure 2D). Also, when we analyzed HA levels in conditioned medium (CM) from tMSLCs and GBM cells, HA levels were higher in CM of tMSLCs than that of GBM cells (Figure 2E). However, HAS2 depletion significantly decreased HA levels in CM of tMSLCs, whereas HAS1 or HAS3 had no such effect (Figure 2F). Although coculture of tMSLCs with X01 GBM cells slightly increased HAS2 levels (Figure 2G), its contribution to HA level was not significantly observed by enzyme-linked immunosorbent assay (ELISA) (Figure 2E). Collectively, these findings indicate that tMSLCs contribute to HA-rich ECM composition through HAS2 in GBM microenvironment.

### Complement factor C5a induces HAS2 in an autocrine manner

**Figure 3:**
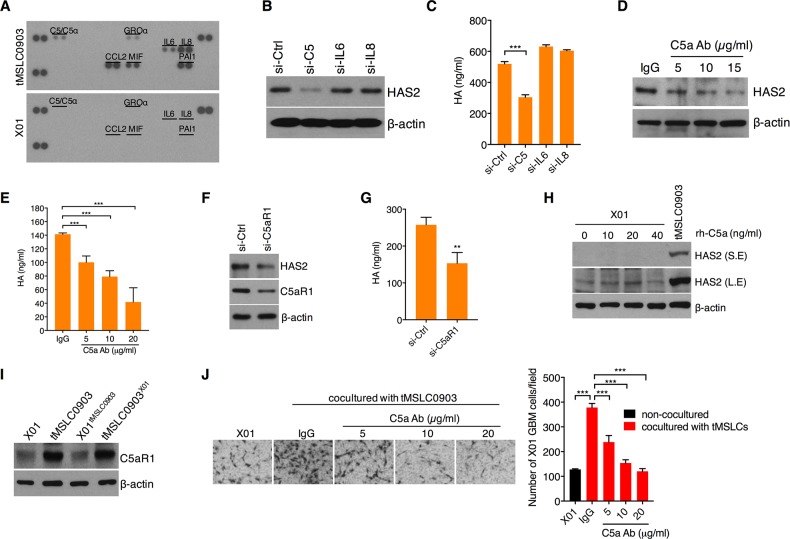
C5a induces HAS2 through C5aR1 in tMSLCs **A.** Cytokine array in tMLSCs or X01 GBM cells. **B.** Western blot analysis for HAS2 in tMSLC0903 after treatment with siRNA as indicated. **C.** ELISA for HA levels in tMSLC0903 after treatment with siRNA as indicated. **D.** Western blot analysis for HAS2 in tMSLC0903 after treatment with C5a antibody. **E.** ELISA for HA levels in tMSLC0903 after treatment with C5a antibody. **F.** Western blot analysis for HAS2 in tMLSC0903 after treatment with siRNA against C5aR1. **G.** ELISA for HA in tMSLC0903 after treatment with siRNA against C5aR1. **H.** Western blot analysis for HAS2 in X01 GBM cells after treatment with rh-C5a. HAS2 in tMLSC0903 is for positive control. S.E., short exposed; L.E., long exposed. **I.** Western blot analysis for C5aR1 in X01, tMSLC0903 or in coculture conditions. **J.** Migration assay of X01 GBM cells alone or cocultured with tMSLCs in the presence of C5a neutralizing antibody. Data are presented as mean ± SD from one of three independent experiments performed. *, p < 0.05 vs. control; **, p<0.01 vs. control; ***, p < 0.001 vs. control.

We next examined a signaling mechanism underlying HAS2 regulation in tMSLCs. By previous studies, because *HAS2* transcription was known to be mainly increased by cytokines [20, 21], we analyzed cytokines that are able to increase *HAS2* in tMSLCs. By cytokine array, we noticed the differential levels of interleukin (IL)-6, IL-8 and C5a between tMSLCs and X01 GBM cells (Figure 3A). When each factor was depleted by siRNA, we found that C5 is necessary for HAS2 induction in tMSLCs (Figure 3B). In agreement with this result, siRNA-mediated C5 depletion effectively diminished HA production in tMSLCs, while depletion of IL-6 or -8 had no such effect (Figure 3C). Likewise, we examined HA and HAS2 levels after treatment with C5a neutralizing antibody. Consistent with siRNA, inhibition of C5a with the antibody also decreased HA as well as HAS2 levels in a dose dependent manner (Figure 3D, 3E). Given that C5a secreted by tMSLCs induces HAS2 expression through its cognate receptor C5aR1, we next depleted C5aR1 with siRNAs in tMSLCs and examined HA and HAS2 levels. As expected, C5aR1 knock down caused a decrease in HAS2 and HA levels in tMSLCs (Figure 3F, 3G).

In GBM cells, however, exogenous addition of recombinant human C5a protein (rh-C5a) did not enhance HAS2 levels, implicating that GBM cells are intrinsically different from tMSLCS in terms of responsiveness to C5a (Figure 3H). Accordingly, we sought to compare levels of C5aR1 between tMSLCs and GBM cells. Importantly, we found that C5aR1 is highly expressed in tMSLCs, while its expression level was substantially lower in GBM cells, providing an explanation for why HAS2 is not induced by treatment with rh-C5a in GBM cells (Figure 3I). We also examined the effect of coculture with tMSLCs; however, it did not enhance C5aR1 level in X01 GBM cells (Figure 3I).

In agreement with the above results, treatment with C5a neutralizing antibody abolished the effect of tMSLCs on invasiveness of GBM cells in a dose dependent manner (Figure 3J). Collectively, these findings suggest that C5a acts as a ligand for C5aR1 for HAS2-mediated HA production in tMSLCs.

### C5a induces HAS2 through ERK MAPK activation in tMSLCs

**Figure 4:**
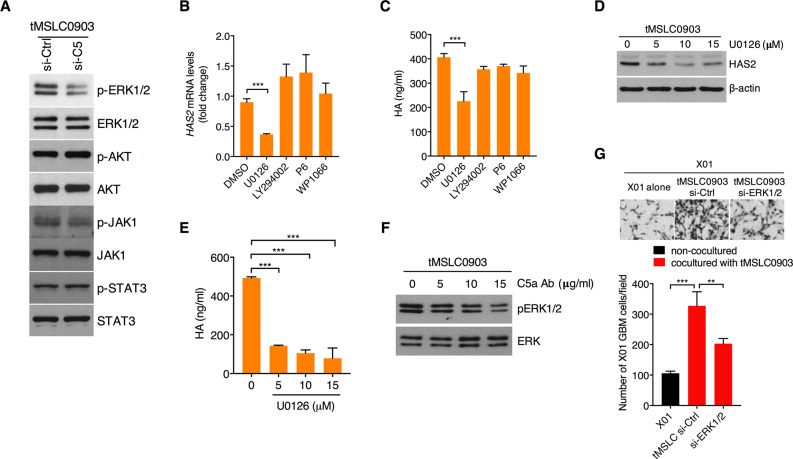
C5a-triggered C5aR1 activation induces HAS2 through ERK MAPK **A.** Western blot analysis for activation status of ERK MAPK, AKT, JAK1 and STAT3 in tMSLCs after treatment with siRNA against C5. **B, C.** qRT-PCR for *HAS2* (B) and ELISA for HA (C) levels after pharmacological inhibition of MEK (U0126), PI3K/AKT (LY294002), JAK1 (P6) or STAT3 (WP1066) in tMSLC0903. **D.** Western blot analysis for HAS2 in tMSLCs after treatment with U0126. **E.** ELISA for HA levels in tMSLC0903 after treatment with U0126. **F.** Western blot analysis for ERK MAPK activation status after treatment with C5a antibody. **G.** Migration assay of X01 GBM cells alone or cocultured with tMSLCs after treatment with siRNA against ERK MAPK. Data are presented as mean ± SD from one of three independent experiments performed. *, p < 0.05 vs. control; **, p<0.01 vs. control; ***, p < 0.001 vs. control.

We next sought to define a signaling mediator in tMSLCs for HAS2 induction in response to C5a. To this end, we examined, after knockdown of C5 in tMSLCs, activation status of several signaling components including ERK, PI3K, JAK and STAT3 that are critical regulators for mediating a variety of cellular phenomena. Treatment with C5 siRNA exclusively inhibited ERK MAPK among them (Figure 4A). In parallel, when ERK signaling is blocked by treatment with MEK inhibitor U0126, qRT-PCR analysis revealed that HAS2 transcript was decreased in tMSLCs, while other inhibitors against PI3K, JAK or STAT3 had no such effect (Figure 4B). Consistent with these results, inhibition of ERK only caused a decrease in HA production in tMSLCs (Figure 4C). To validate that ERK is a critical regulator for HA production, we treated tMSLCs with a various concentration of MEK inhibitor U0126 (0-15 μM), and analyzed HAS2 and HA levels. Consistent with the above data, treatment with U0126 also decreased HAS2 and HA levels in tMSLCs in a dose dependent manner (Figure 4D, 4E). In agreement with these results, treatment with neutralizing C5a antibody inhibited phosphorylation of ERK1/2 (Figure 4F). In parallel, ERK depletion in tMSLCs blocked the coculture effect on migration of X01 GBM cells (Figure 4G). Taken together, these results suggest that C5a induces HAS2 through ERK MAPK activation in tMSLCs.

### HA promotes invasiveness of GBM cells by acting as a ligand for RHAMM receptor

**Figure 5:**
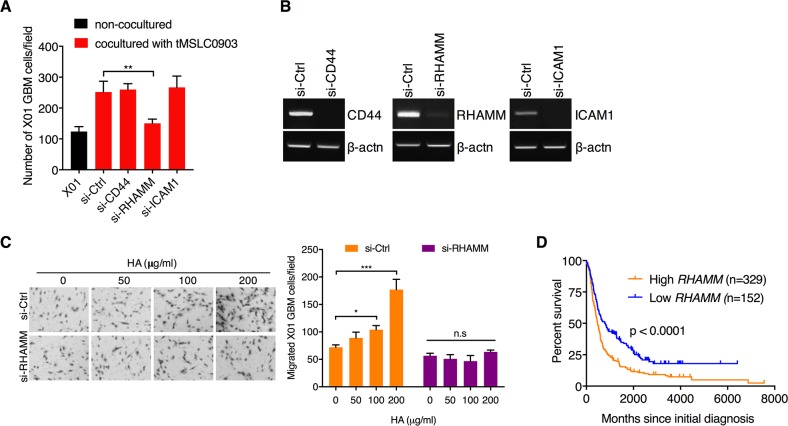
HA abundance promotes migration of GBM cells through RHAMM receptor **A.** Migration assay of X01 GBM cells alone or cocultured with tMSLCs after treatment with siRNA as indicated. **B.** Semi-quantitative RT-PCR for validation of siRNA efficacy. **C.** Migration assay of X01 GBM cells that are transfected with siRNA against *RHAMM* and treated with various concentration of HA. **D.** Kaplan-Meier survival curves of patients with brain tumors in high and low levels of *RHAMM*. Data are presented as mean ± SD from one of three independent experiments performed. *, p < 0.05 vs. control; **, p<0.01 vs. control; ***, p < 0.001 vs. control.

Previous studies have reported that many of the effects of HA are mediated through cell surface receptors, three of which have been molecularly characterized: CD44, RHAMM, and ICAM-1 [22]. To understand how HA-rich microenvironment facilitates invasiveness of GBM cells, we treated tMSLCs with siRNA against each HA receptor and analyzed the coculture effect of tMSLCs on invasiveness of GBM cells. By migration analysis in Transwell, we found that RHAMM, among them, is critical for the effect of tMSLCs on migration of GBM cells (Figure 5A, 5B). Consistent with this finding, RHAMM depletion significantly abolished the effect of exogenous HA addition on invasiveness of GBM cells (Figure 5C). In addition, we evaluated whether RHAMM levels in human brain tumor are correlated with patient survival. Evaluation of data in REMBRANDT database revealed that expression levels of *RHAMM* are inversely correlated with the survival of patient with brain tumor (Figure 5D). Taken together, these results indicate that HA promotes phenotypic change of GBM cells into invasiveness through RHAMM.

### Targeting C5a impedes *in vivo* infiltration of GBM cells

**Figure 6:**
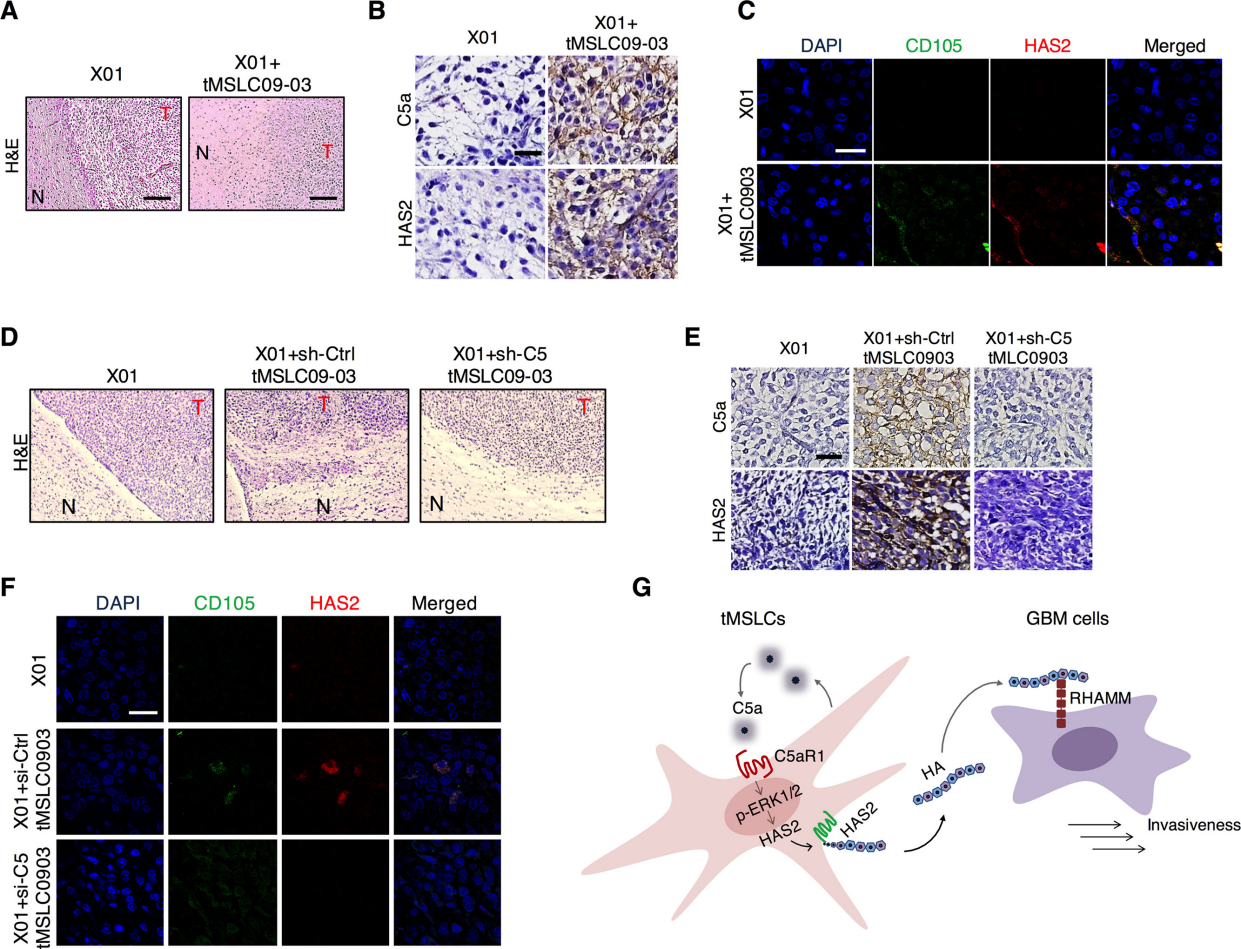
Correlation of C5a with HAS2 levels in tumor microenvironment **A.** H&E staining in coronal-sectioned GBM tumors formed by implantation of X01 GBM cells alone or with tMSLCs into mouse brain. Scale bar, 500 μm. **B.** Immunohistochemical staining of C5a and HAS2 in the orthotopic xenograft GBM tumors formed by implantation of X01 GBM cells alone or with tMSLCs into mouse brain. Scale bar, 20 μm. **C.** Immunocytochemical staining showing colocalization of HAS2 with CD105 in the orthotopic xenograft GBM tumors. Scale bar, 20 μm. **D.** H&E staining in coronal-sectioned GBM tumors formed by implantation of X01 GBM cells alone or with tMSLCs into mouse brain. tMSLCs were transfected with siRNA as indicated, prior to xenograft. Scale bar, 500 μm. **E.** Immunohistochemical staining of C5a and HAS2 in the orthotopic xenograft GBM tumors formed by implantation of X01 GBM cells alone or with tMSLCs into mouse brain. tMSLCs were transfected with siRNA as indicated, prior to xenograft. Scale bar, 20 μm. **F.** Immunocytochemical staining showing colocalization of HAS2 with CD105 in the orthotopic xenograft GBM tumors. Scale bar, 20 μm. **G.** Schematic model illustrating HA production in tMSLCs that enhances invasiveness of GBM cells in tumor microenvironment. T, tumor; N, normal.

To validate our findings *in vivo*, we orthotopically implanted X01 GBM cells alone or with tMSLCs into mouse brain. In agreement with *in vitro* data, we found that GBM cells exhibited more infiltration into parenchymal brain following tumor formation when co-injected with tMSLCs, as evidenced by histological staining in which tumor margin appears to be less distinctive and GBM cells are likely to diffuse more to normal tissue, compared with tumor formed by GBM cells alone (Figure 6A). An immunohistochemical analysis also revealed higher levels of C5a and HA in tumors formed by X01 GBM cells combined with tMSLCs than in tumors formed by X01 GBM cells alone (Figure 6B). In addition, Immunocytochemical staining revealed colocalization of HAS2 with MSC marker CD105 in the xenograft tumors, supporting that HAS2 is expressed preferentially in tMSLCs (Figure 6C).

To confirm the role of C5a *in vivo*, we also transduced tMSLC09-03 with C5 shRNA or scrambled control shRNA prior to orthotopic co-inoculation with X01 GBM cells into mouse brains. GBM tumors were formed ~5 weeks after implantation of GBM cells. As expected, GBM cells co-implanted with tMSLCs infiltrated more into normal brain parenchyma than GBM cells implanted alone; however, C5 depletion in tMSLCs attenuated the effect on GBM cell infiltration (Figure 6D). In addition, C5 depletion diminished the effect of tMSLCs on HA production *in vivo*, as evidenced by immunohistochemical staining (Figure 6E). Again, we observed the colocalization of HAS2 with MSC marker CD105 (Figure 6F). Collectively, our findings suggest that tMSLCs contribute to abundance of HA in GBM tumor microenvironment, and thereby enhances invasiveness of GBM cells as depicted in Figure 6G.

## DISCUSSION

Although a long-standing focus has been in cell-autonomous mechanisms to understand cancer progression, paracrine crosstalk between tumor cells and stromal cells has begun to be recognized as important contributors to cancer progression. In line with this trend, tMSLCs are recently defined as stromal cells interacting with GBM cells and their potential role in tumor progression has received intensive attention [11, 12, 23–25].

Given that tMSLCs were previously isolated from human GBM surgical specimens and their presence is closely correlated with the prognosis of patients [12], we further analyzed the effect of tMSLCs on invasiveness of GBM cells. In this current study, we found that tMSLCs act as a booster for infiltration of GBM cells by providing HA-rich ECM composition in tumor microenvironment. By previous studies, the abundance of HA in tumor has been proportional to invasiveness of tumor cells [9]. Moreover, high levels of HA in stroma are associated with poor prognosis of the patients [10]. Despite its clinical importance, however, its mechanisms underlying the abundance of HA in GBM region remain unexplained. Notably, our findings demonstrate that HA abundance in tumor microenvironment could be caused by tMSLCs. We showed that C5a secreted from tMSLCs activates ERK MAPK for HAS2 induction in an autocrine manner, thereby increases HA production. Supporting our data, previous study has shown that ERK phosphorylation is prolonged by C5a in mesenchymal stem cells [26]. In agreement, our study showed that targeting of C5a effectively decreases ERK MAPK activation and HAS2 level in tMSLCs, resulting in lowering HA levels.

C5a is well known as a complement component that triggers degranulation of mast cells or neutrophils, enhancing their phagocytosis of pathogens [18, 27]. However, in addition to its primary functions, the new functions of C5a in cancer have begun to gain attention. Although complement system has been thought traditionally to have a role for immune surveillance, several strong lines of evidence demonstrated that complement factors including C5a contribute to cancer progression [27–29]. The involvement of C5a in promoting carcinogenesis and metastasis is explained by the recruitment of myeloid-derived suppressor cells (MDSCs) and the generation of an immunosuppressive microenvironment [30]. Our finding that C5a acts as a cue for proinvasive tumor microenvironment is in line with these previous reports.

As an ECM component, HA was known to act as a permissive role in enhancing the invasiveness of GBM cells by providing mechanical matrix in tumor microenvironment [5, 31]. In addition, HA also acts as a signaling component that transduces intracellular signaling pathways for invasiveness of GBM cells through its cognate receptors such as CD44, RHAMM, and ICAM-1 [5, 7, 8]. Previously, many studies have shown the importance of those HA receptors in GBM progression [32–34]. In this current study, our findings revealed that HA-rich microenvironment promotes phenotypic changes of GBM cells into invasiveness through RHAMM receptor, implicating the importance of RHAMM as a HA receptor in GBM cells. However, since it was observed only in one patient-derived GBM cells, our finding does not mean that the effect of HA occurs exclusively through RHAMM in GBM cells. In our thoughts, HA may transduce intracellular signaling through the cognate receptor, depending on status of GBM cells such as the receptor levels on membrane.

In summary, we demonstrate that tumor-associated MSLCs are responsible for HA abundance in GBM microenvironment and act as a booster for invasiveness of GBM cells. Considering the effect of HA on survival of GBM patients, the mechanisms underlying tMSLC-mediated HA production merits further investigation.

## MATERIALS AND METHODS

### Chemical reagents and antibodies

Antibodies to p-ERK1/2, ERK1/2, p-T308-AKT, AKT, p-Y705-STAT3, JAK1 were purchased from Cell Signaling Technology (Beverly, MA, USA). STAT3 and p-T1022-JAK1 were purchased from Santa Cruz Biotechnology (Santa Cruz, CA, USA). Antibodies to HAS2 and C5R1 were purchased from Abcam (Cambridge, UK). 4,6-diamidino-2-phenylindole (DAPI) were purchased form Sigma (St Louis, MO, USA). rh-C5a protein and antibodies to CD105 and C5a were purchased from R&D Systems (Minneapolis, MN, USA). Chemical inhibitors specific to MEK (U0126), PI3K (LY294002), JAK1 (P6) and STAT3 (WP1066) were purchased from Calbiochem (San Diego, CA, USA). Anti-Goat Alexa Fluor 488, anti-mouse Alexa Fluor 546 were purchased from Invitrogen (Carlsbad, CA, USA). Anti-mouse IgG-HRP, anti-goat IgG-HRP and anti-rabbit Ig-HRP were purchased from Santa Cruz biotechnology (Santa Cruz, CA, USA). HA (Low molecular weight; 15-40 kDa) was purchased from R&D systems (Minneapolis, MN, USA).

### Co-culture of tMSLC and GBM cells

tMSLC0903 (2.5 × 10^5^) were seeded onto the upper chamber and X01 GBM cells (1.5 × 10^5^) into the lower chamber in Transwell with a pore size 0.4 μm. Migration was analyzed 3 days after co-culture.

### Culture of GBM cells and tMSLCs

The patient-derived X01 GBM cells were established from acutely resected human tumor tissues obtained with written informed consent from a 68-year-old woman with GBM [35]. X01 GBM cells were cultured in Dulbecco's modified Eagle Medium (Gibco, Korea, Seoul) containing 1% penicillin and streptomycin, supplemented with 10% heat-inactivated fetal bovine serum (Lonza, Basel, Switzerland).

tMSLC09-03 (previously referred as KGS-MSC05-04) have been isolated from biopsy of GBM patient [23]. All non-adherent cells were washed away with phosphate-buffered saline (PBS) and only adherent tMSLCs were cultured in MEMα (Mediatech) containing 10% FBS (Lonza), 2 mM L-glutamine (Mediatech), and antibiotic–antimycotic solution (Gibco, Seoul, Korea).

### Migration assay

Migration properties of GBM cells were analyzed in transwell (8 μm pore size; Corning Glass, Seoul, Korea). GBM cells (2 × 10^4^) were loaded in the upper well of the transwell and were incubated for 24 h. The migrated cells into the lower surface of the filter were then fixed and stained with a Diff Quick kit (Fisher, Pittsburgh, PA, USA). The number of migrated cells was counted in three microscopic fields per well.

### Organotypic invasion of GBM cells in ECM conditioned by tMSLCs

tMSLC0903 were seeded into the collagen-based matrix, and cultured for 3 days for tMSLC0903-caused ECM remodeling. Afterwards, tMSLC0903 were killed by treatment with puromycin, but leaving tMSLC-secreted ECM composition. X01 GBM cells were then plated in the conditioned ECM, and their invasion was visualized at 48 h by H&E staining after perpendicular section of the gels. The number of invaded cells was counted in three microscopic fields per well.

### Cytokine array

The human cytokine array (Proteome Profiler Array Human Cytokine, R&D Systems, ARY005, Minneapolis, MN, USA) was performed according to the manufacturer's instructions. Those cytokine levels were visualized in the X-ray film and quantified by densitometry using Image J software.

### ELISA

CM was collected 3 days after plating X01 GBM cells or tMSLCs, and the secreted HA in CM was quantified by ELISA (human HA ELISA Kit, R&D Systems) according to the manufacturer's protocol.

### Transfection

Cells were transfected with siRNA using a Microporator-mini (Digital Bio Technology, Seoul, Korea) according to the procedure recommended by the manufacturer. All siRNAs were purchased from Genolution Pharmaceuticals, Inc (Seoul, Korea).

### Western blot analysis

Cell lysates were prepared by extracting proteins with lysis buffer [40 mM Tris–HCl (pH 8.0), 120 mM NaCl, 0.1% Nonidet-P40] supplemented with protease inhibitors. Proteins were separated by SDS-PAGE, and transferred to a nitrocellulose membrane (Amersham, Arlington Heights, IL, USA). The membrane was blocked with 5% non-fat dry milk in Tris-buffered saline, and incubated with primary antibodies overnight at 4°C. Blots were developed with a peroxidase-conjugated secondary antibody, and proteins visualized by enhanced chemiluminescence (ECL) procedures (Amersham), using the manufacturer's protocol.

### RT-PCR

Total RNA was isolated using the Trizol (Invitrogen). RNA was subjected to reverse transcription using SuperScript III First-Strand Synthesis SuperMix (Invitrogen). Gene expression levels were analyzed by PCR using Econo Taq Plus Green (Lucigen, Seoul, Korea). β-actin was used as an internal control for each sample.

### Immunohistochemistry

Mouse tissues were fixed in formalin for the preparation of paraffin sections. Paraffin-embedded tissue sections were deparaffinized in xylene, 95, 90, and 70% ethanol, followed by PBS. Epitopes were unmasked with 20 mg/mL proteinase K in PBS with 0.1% Triton X-100. Sections were stained with H&E or immunostained overnight at 4°C with primary antibody. After washing in PBS, biotinylated goat anti-rabbit IgG or anti-mouse IgG antibody was then applied to the sections for 30 min. After washing in PBS, ABC reagent (Vector Laboratories Inc, Burlingame, CA, USA) was applied to the sections for 30 min. Color reaction was performed with 3, 3′-diaminobenzidine (Vector Laboratories). After counter-staining with hematoxylin and clearing with graded ethanol series and xylene, the sections were mounted with Canada balsam.

Alternatively, tissues were incubated with primary antibody at 4°C overnight. Samples were then visualized by staining with anti-mouse Alexa Flour488 and anti-goat Alexa Flour546 (Molecular Probes, Seoul, Korea). Nuclei were counterstained using DAPI (Sigma, St. Louis, MO, USA). Images were captured with Nikon confocal microscope C2 installed at the Hanyang Center for Research Facilities (Seoul), and analyzed with NIS-Elements Viewer 4.2 (Nikon Instruments, Tokyo, Japan).

### Animal studies

5 to 8-weeks-old male athymic nude mice (Central Lab. Animal Inc., Seoul, Korea) were used to check tumorigenesis of GBM cells and tMSLCs. Mice were housed in microisolator cages under sterile conditions and observed for at least 1 week before study initiation to ensure proper health. GBM cells alone or combined with tMSLC0903 (1:1 ratio) were injected at a speed of 0.5 μl/min into the right frontal lobe of mouse skull via a Hamilton syringe (Dongwoo Science Co., Seoul, Korea) using a guide-screw system, as described previously [23, 36, 37].

### Kaplan-meier survival analysis

The National Cancer Institute's Repository for Molecular Brain Neoplasia Data (REMBRANDT, http://www.betastasis.com/glioma/rembrandt, accessed November 2016) was evaluated for correlations between clinical outcome/survival and *RHAMM* gene expression in human brain tumor biopsies. For REMBRANDT, “high expression” is defined as upper than threshold 6.31; “low expression” is defined as lower than the threshold.

### Statistical analysis

All experimental data are reported as means; error bars represent standard deviation (SD). Comparisons between values were performed using unpaired two-tailed Student's t-test, or ANOVA for multivariate analysis. All statistical analyses were performed using GraphPad Prism 7.0 and the *p* values <0.05 were considered significant.
